# Preliminary exploration of hepatic parenchymal near-infrared fluorescence imaging technique via retrograde biliary approach: a feasibility study (with video)

**DOI:** 10.1038/s41598-024-52904-9

**Published:** 2024-01-29

**Authors:** Fengwei Gao, Qingyun Xie, Xin Zhao, Manyu Yang, Kangyi Jiang, Ling Zhang, Tianyang Mao, Hong Wu

**Affiliations:** 1 Liver Transplantation Center, State Key Laboratory of Biotherapy and Cancer Center, West China Hospital, Sichuan University and Collaborative Innovation Center of Biotherapy, No. 37, Guoxue Lane, Wuhou District, Chengdu, 610041 Sichuan Province China; 2 Department of Hepato-Pancreato-Biliary Surgery, The People’s Hospital of Leshan, Leshan, 614000 China; 3 North Sichuan Medical College, Nanchong, 637000 China

**Keywords:** Preclinical research, Diseases

## Abstract

This paper explores the feasibility and principle of hepatic parenteral fluorescence imaging technology after retrograde injection of indocyanine green (ICG) through endoscopic nasobiliary drainage (ENBD). The data were collected from 53 patients with cholecystolithiasis and choledocholithiasis, from October 2022 to March 2023, diagnosed by fluorescence imaging technique retrograde biliary approach (FIT-RB). We divided the patients into two groups according to the features of liver parenchyma, the poor group (n = 34, including scattered or no imaging) and the good group (n = 19, regular uniform imaging). We compared and analyzed the perioperative results of the two groups and explored the influencing factors of the success of FIT-RB and the ICG concentration suitable for this imaging technique. The good imaging rate of the 53 enrolled cases was 35.8%. The bilirubin level before ENBD and laparoscopic cholecystectomy in the poor group was significantly higher than that in the good group (*P* < 0.001). The proportion of higher ICG concentrations (0.5 mg/mL) was significantly higher in the good group (*P* = 0.028). Our results demonstrated that the success rate of good imaging was 4.53 times higher than that of low-dose ICG (0.125 or 0.25 mg/L) cases at 0.5 mg/ml of ICG. The level of total bilirubin and direct bilirubin were negatively correlated with the imaging effect, and total bilirubin and direct bilirubin levels were important predictors of the efficacy of FIT-RB. FIT-RB is safe and feasible in patients with low site bilirubin levels. An ICG concentration of 0.5 mg/ml may be ideal for implementing this technique.

## Introduction

Indocyanine Green (ICG) is a fluorescent dye that exhibits near-infrared spectroscopy and is used in medical imaging and diagnostic procedures^1,2^. Because of its benefits, such as non-radiation, high safety profile, and minimal impact on liver function, it is widely used in assessing liver reserve function, diagnosing liver diseases, and guiding intraoperative navigation during liver surgery^3,4^. For navigation during anatomic hepatectomy (AR), the most widely used field of ICG, it mainly relies on “positive” or “negative” staining techniques to achieve fluorescence visualization of liver segments or lobes via portal vein^5,6^. Currently, various combined intervention techniques are also utilized to achieve fluorescence visualization, such as the “positive dyeing” technique via the hepatic artery^7–10^. Additionally, in the surgical treatment of intrahepatic bile duct calculi, some scholars have utilized the principle of ICG excretion through a biliary duct to accurately locate the scope of atrophic liver, intrahepatic calculi, and diseased bile duct,^11,12^. Following the intraoperative injection of ICG, immediate fluorescence imaging could not be attained due to hindrance caused by obstruction of bile excretion in the liver tissue set for resection. Consequently, a navigation effect resembling the “negative staining” technology could be observed^13^. However, in some cases, although there are intrahepatic bile duct stones and liver atrophy, the stenosis of the bile duct is incomplete. It is difficult for the bile containing ICG molecules to accumulate in the diseased bile duct and display fluorescence. To overcome this problem, in a case of laparoscopic anatomical S3 resection for local intrahepatic bile duct stones, after blocking the bile duct of segment III, we used endoscopic nasobiliary drainage (ENBD) to inject ICG, thus enabling the normal liver parenchyma to fluoresce, achieving a “reverse staining” effect to guide the anatomical resection of the entire diseased bile duct tree (Video S4).

The primary objective of this study is to investigate the feasibility and imaging principles of this newly discovered fluorescence imaging technique for visualizing liver parenchyma. The technique involves injecting the fluorescent dye indocyanine green (ICG) into the biliary tract via an endoscopic nasobiliary drainage tube (ENBD) in a retrograde manner. The dye then accumulates in normal liver tissue, allowing for visualization using near-infrared laparoscopic imaging systems. This retrograde biliary approach to fluorescence imaging of the liver parenchyma holds potential advantages in guiding anatomical liver resections for patients with hepatolithiasis. However, the feasibility and imaging principles of this innovative technique require further evaluation.To address these objectives, we conducted an exploratory clinical study aiming to: 1. Demonstrate the safety and feasibility of retrograde biliary ICG injection for fluorescence imaging of liver parenchyma. 2. Elucidate the imaging principles and patterns associated with this technique.3. Identify factors that influence successful fluorescence imaging.

## Methods

In this study, samples from 53 patients, diagnosed with both cholecystolithiasis and choledocholithiasis were collected and statistically analyzed by fluorescence imaging technique retrograde biliary approach (FIT-RB) from October 2022 to March 2023. According to the imaging findings of hepatic parenchyma, the patients were divided into the poor group (n = 34, including scattered or no imaging) and the good group (n = 19, regular and uniform imaging). The perioperative results of the two groups were compared and analyzed, and the factors influencing the success of FIT-RB and the ICG concentrations suitable for this imaging technique were discussed.

Inclusion criteria: (1) Preoperative diagnosis of cholecystolithiasis combined with choledocholithiasis, established through patient history and abdominal imaging^14^; (2) Consistent with symptoms of laparoscopic cholecystectomy^15^; (3) adherence to the indication for endoscopic treatment of choledocholithiasis; (4) Patients with a history of previous abdominal surgery; (5) Absence of underlying cardiopulmonary dysfunction; (6) No history of hepatitis or liver disease caused by various factors. Exclusion criteria: (1) Incomplete clinical data (including intraoperative video); (2) Allergic to ICG; (3) Consideration of patients with potential gallbladder or biliary malignancy; (4) Patients with incomplete removal of calculi during endoscopy; (5) Patients with severe postoperative complications of ERCP; (6) Patients with dysfunction of other vital organs.

This prospective feasibility study received approval from the Medical Ethics Committee on Biomedical Research, West China Hospital of Sichuan University [Ethics number: 2021 Review (1682)]. The study was performed in accordance with the Declaration of Helsinki.

### Research process

**Figure 1:**
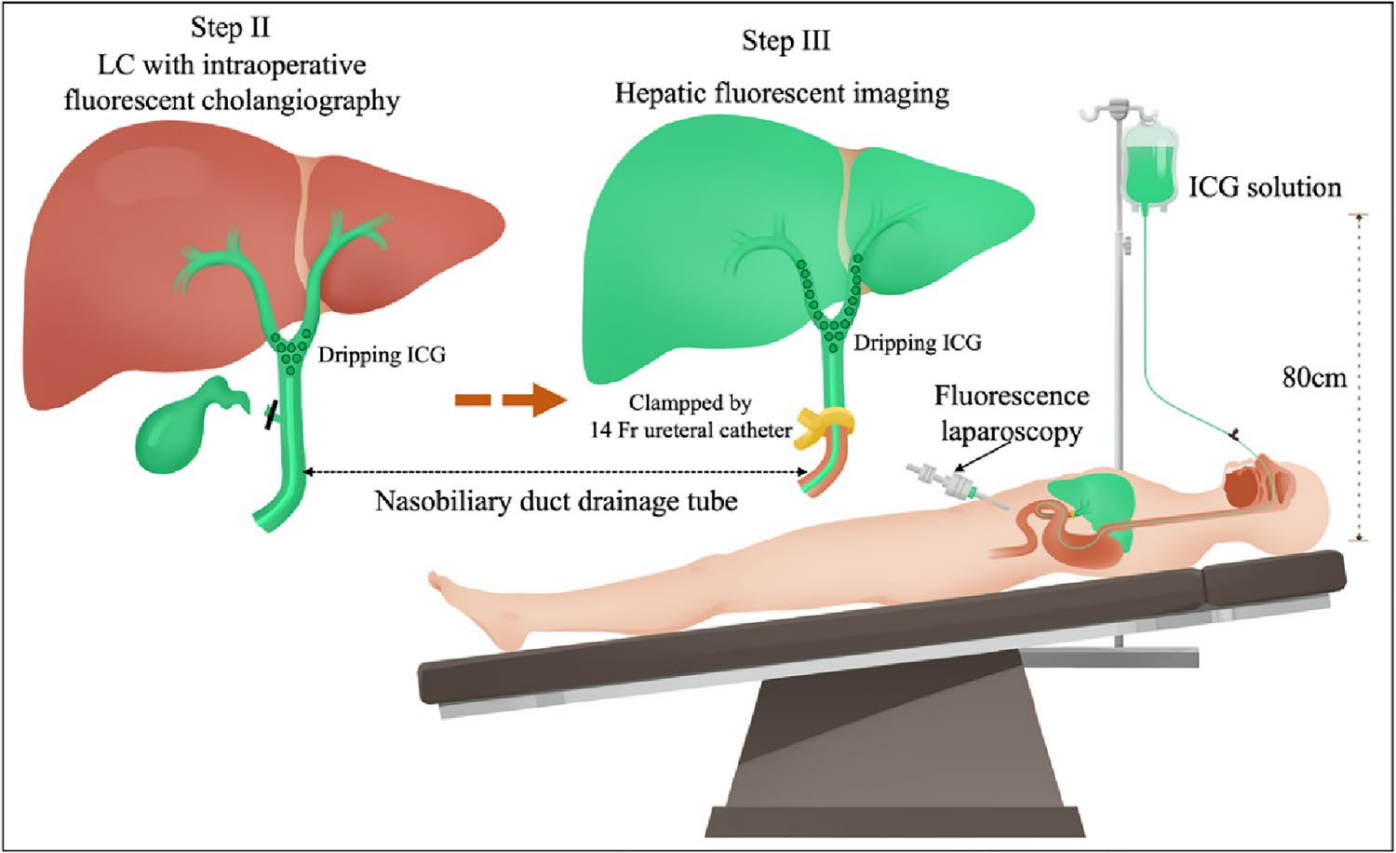
Schematic of hepatic parenchymal fluorescent imaging technique via retrograde biliary approach.

The procedure of this study was divided into three stages. In the first stage, choledocholithiasis was removed by ERCP+ endoscopic papillary balloon dilatation (EPBD) + ENBD technique. The second stage involved the utilization of laparoscopic fluorescence imaging systems (Digital Precision Medical Technology Co., Ltd, the day after ERCP surgery, In China), near-infrared fluorescent-guided laparoscopic cholecystectomy (LC) was performed, and various concentrations of ICG (DiagnoGreen^®^, Taiwan, ROC) solution were injected into ENBD tube in vitro for Diagnogreen cholecystectomy. In the third stage, hepatic pedicle occlusion was performed using a No. 14 catheter after cholecystectomy, while ICG solution was continued to be injected, and fluorescence mode was turned on to observe the fluorescence imaging effect of the liver within a 10-min blocking cycle (Fig. 1).

**Figure 2:**
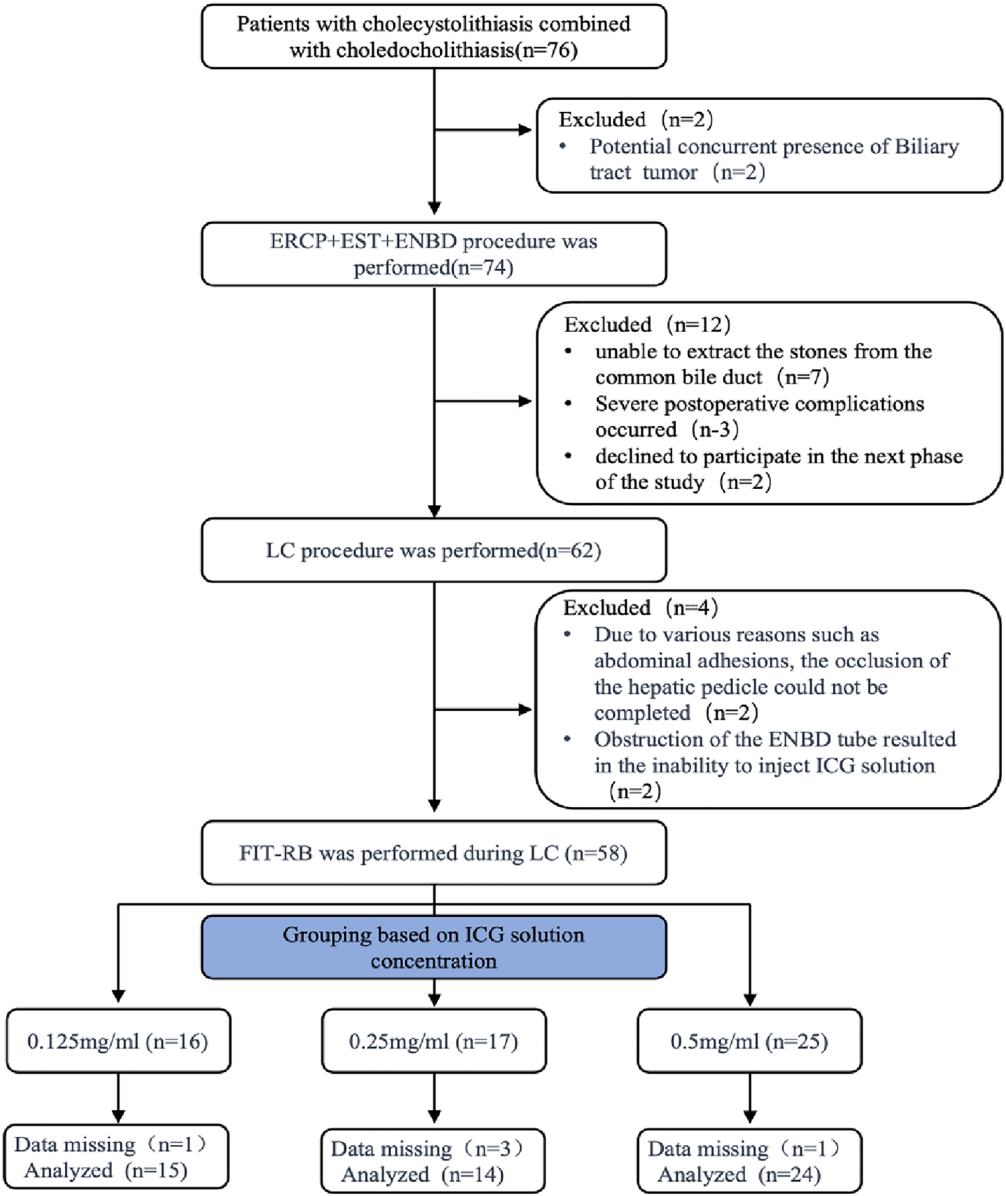
Flow chart for selecting patients for this study.

The study was conducted from October 2022 to March 2023. A total of 76 subjects initially signed informed consent for this prospective study, and 53 cases (15 + 14 + 24) with complete clinical data (including intraoperative videos) were finally included in the analysis (Fig. 2). According to the quality of liver parenchyma imaging, the two groups were divided into the poor imaging group (n = 34, including scattered or no imaging) and the good imaging group (n = 19, regular uniform imaging).

### Statistical analysis

Quantitative data were presented as means ± standard deviation and median (Q1–Q3). Descriptive statistics for qualitative data utilized a frequency (%) representation. Before analysis, all measurement data were subjected to a Kolmogorov–Smirnov normality test. As long as the data conformed to the normal distribution, the t-test was used to determine whether there was a significant difference between the two groups; otherwise, the Wilcoxon rank-sum test was applied. Qualitative data were analyzed using the chi-square test to determine the significance of differences between groups.

Univariate logistic regression analyses were performed to identify the variables independently associated with imaging.

ROC curve analysis was employed to assess the indicators with statistical significance. The area under the ROC curve (AUC) was calculated to evaluate the ability of these factors to predict imaging results. Statistical analysis was performed with R version 4.0.3 (https://www.R-project.org) and Easy R (https://www.easyr.cc Solutions, Inc., Shanghai). A two-tailed *P* value of < 0.05 was considered statistically significant in all analyses.

### Ethical approval and informed consent

This study was prospectively conducted with the consent of the Ethics Committee of our hospital (Ethics number: 2021 Review (1682)) and with informed consent obtained from the subjects. All methods were carried out in accordance with relevant guidelines and regulations. Research involving human participants, human material, or human data was conducted in accordance with the Declaration of Helsinki. The informed consent was obtained from all subjects. This prospective feasibility study was approved by the Medical Ethics Committee on Biomedical Research, West China Hospital of Sichuan University (Ethics number: 2021 Review (1682)).

## Results

Table 1 summarizes the demographic, clinical, and biochemical characteristics of the patients. Based on the results of imaging, the patients were divided into two groups. There were no significant differences between the two groups in terms of demographic information, including age, sex, and BMI. However, we found that the good group had lower levels of ENBD-TBIL (total bilirubin level), ENBD-DBIL (direct bilirubin level), LC-TBIL, and LC-DBIL. The proportion of higher ICG concentrations (0.5 mg/ml) was significantly higher in the good group (*P* = 0.028). There were no statistically significant differences in the diameter of the bile duct or the presence of fatty liver between the two groups.

**Table 1:** Description of population characteristics.

Imaging	Poor group (n = 34)	Good group (n = 19)	*P* value
Age (year)	53.00 (36.50–62.50)	57.00 (49.00–69.00)	0.124
BMI (kg/m^2^)	21.35 (19.33–23.40)	22.10 (19.40–22.90)	0.817
Choledochal diameter (cm)	1.05 (0.90–1.30)	1.10 (0.90–1.25)	0.815
ENBD-TBIL (umol/L)	41.65 (21.57–81.67)	18.50 (12.40–27.70)	0.004
ENBD-DBIL (umol/L)	30.75 (13.50–60.52)	12.50 (4.30–19.05)	0.003
LC-TBIL (umol/L)	33.75 (22.57–62.07)	17.90 (12.75–20.70)	< 0.001
LC-DBIL (umol/L)	19.05 (12.53–40.70)	9.10 (7.70–12.10)	< 0.001
Gender			0.581
1 (Male)	17 (50.00%)	8 (42.11%)	
2 (Female)	17 (50.00%)	11 (57.89%)	
Fatty liver			0.518
0 (No)	21 (61.76%)	10 (52.63%)	
1 (Yes)	13 (38.24%)	9 (47.37%)	
ICG concentration			0.028
0.125 mg/ml	13 (38.24%)	2 (10.53%)	
0.25 mg/ml	10 (29.41%)	4 (21.05%)	
0.5 mg/ml	11 (32.35%)	13 (68.42%)	

*ENBD-TBIL* total bilirubin level before endoscopic nasobiliary drainage, *ENBD-DBIL* direct bilirubin level before endoscopic nasobiliary drainage, *LC-TBIL* total bilirubin level before laparoscopic cholecystectomy, *LC-DBIL* direct bilirubin level before laparoscopic cholecystectomy, *ICG* indocyanine green, *BMI* body mass index.

**Figure 3:**
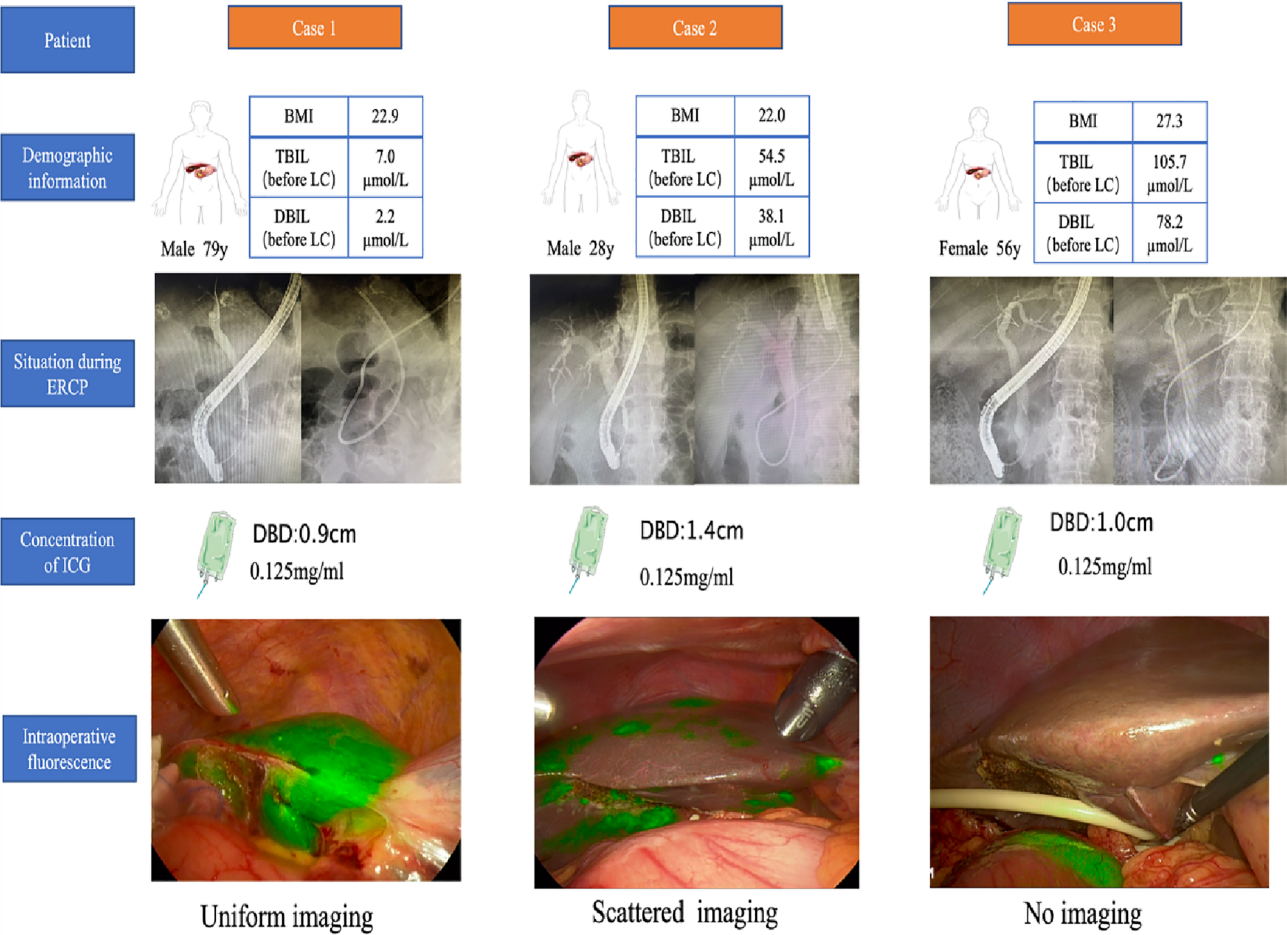
The fluorescence imaging effects of liver parenchyma in case 1, case 2, and case 3 after injection of 0.125 mg/ml ICG solution through ENBD tube were evaluated as Uniform imaging, Scattered imaging, and No imaging, respectively. Diameter of the bile duct (DBD): diameter of the bile duct.

Fluorescence enhancement effect decision criteria: In this study, all conducted operations on all patients were recorded, and the videos were analyzed by experts. Ultimately we categorized the observed liver parenchyma into three types of fluorescence effects, as illustrated in Fig. 3: (Uniform imaging, Scattered imaging, and No imaging).

The results of univariate analysis for the demographic and clinical characteristics of the study participants are presented in Table 2. The results showed that age, sex, BMI, and diameter of the bile duct were not significantly associated with imaging. We found that in the group with ICG concentration of 0.5 mg/ml, the proportion of successful/positive contrast was 4.53 times higher than in the low-dose group with ICG. Total bilirubin and direct bilirubin are negatively correlated with the imaging.

**Table 2:** Univariate analysis of the demographic and clinical characteristics of the study participants.

	Statistics	Imaging
Age (year)	53.64 ± 16.26	1.03 (0.99, 1.07) 0.1181
Gender		
1 (Male)	25 (47.17%)	1.0
2 (Female)	28 (52.83%)	1.37 (0.44, 4.26) 0.5814
BMI (kg/m^2^)	21.65 ± 2.88	0.94 (0.77, 1.15) 0.5630
Choledochal diameter (cm)	1.11 ± 0.26	0.98 (0.11, 8.59) 0.9867
Fatty liver		
0 (No)	31 (58.49%)	1.0
1 (Yes)	22 (41.51%)	1.45 (0.47, 4.53) 0.5183
ENBD-TBIL	46.69 ± 43.32	0.97 (0.94, 0.99) 0.0173
ENBD-DBIL	32.63 ± 32.79	0.96 (0.92, 0.99) 0.0176
LC-TBIL	35.73 ± 31.28	0.91 (0.85, 0.98) 0.0082
LC-DBIL	23.02 ± 23.46	0.89 (0.80, 0.98) 0.0156
ICG NEW		
0	29 (54.72%)	1.0
1	24 (45.28%)	4.53 (1.36, 15.12) 0.0140

**Figure 4:**
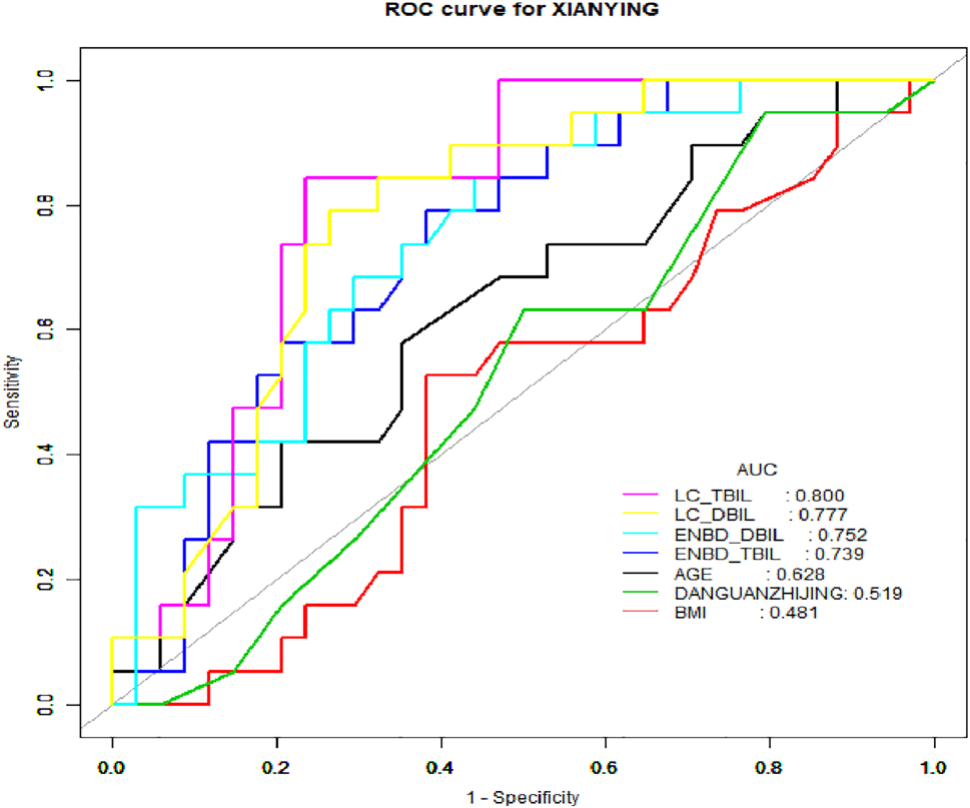
Single factor ROC analysis curve.

The ROC curves for seven potentially predictive factors (LC-TBIL, LC-DBIL, ENBD-DBIL, ENBD-TBIL, age, diameter of bile duct and BMI) were plotted (see Table 3, Fig. 4). The following AUC values for the eight factors were calculated. The predictive factors for imaging were ENBD-TBIL (AUC = 0.7392, 95% CI 0.6046–0.8737), ENBD-DBIL (AUC = 0.7515, 95% CI 0.6185–0.8846) LC_TBIL (AUC = 0.8003, 95% CI 0.6809–0.9198) and LC-DBIL (AUC = 0.7771, 95% CI 0.6512–0.9030). In addition, AGE (AUC = 0.6285, 95% CI 0.4718–0.7852) and Choledochal diameter (AUC = 0.5193, 95% CI 0.3614–0.6773) were also found to be predictive factors, but their predictive power was weaker. These results suggest that ENBD-TBIL, ENBD-DBIL, LC-TBIL, and LC-DBIL are important predictors for FIT-RB.

**Table 3:** Data of single factor ROC analysis curve.

Test	AUC	95% CI low	95% CI upp	Best threshold	Specificity	Sensitivity
AGE	0.6285	0.4718	0.7852	56.5000	0.6471	0.5789
BMI	0.4807	0.3194	0.6419	22.0500	0.6176	0.5263
Choledochal diameter	0.5193	0.3614	0.6773	0.8500	0.2059	0.9474
ENBD-TBIL	0.7392	0.6046	0.8737	30.5500	0.6176	0.7895
ENBD-DBIL	0.7515	0.6185	0.8846	28.1000	0.5294	0.8947
LC-TBIL	0.8003	0.6809	0.9198	22.4000	0.7647	0.8421
LC-DBIL	0.7771	0.6512	0.9030	12.8500	0.7353	0.7895

**Figure 5:**
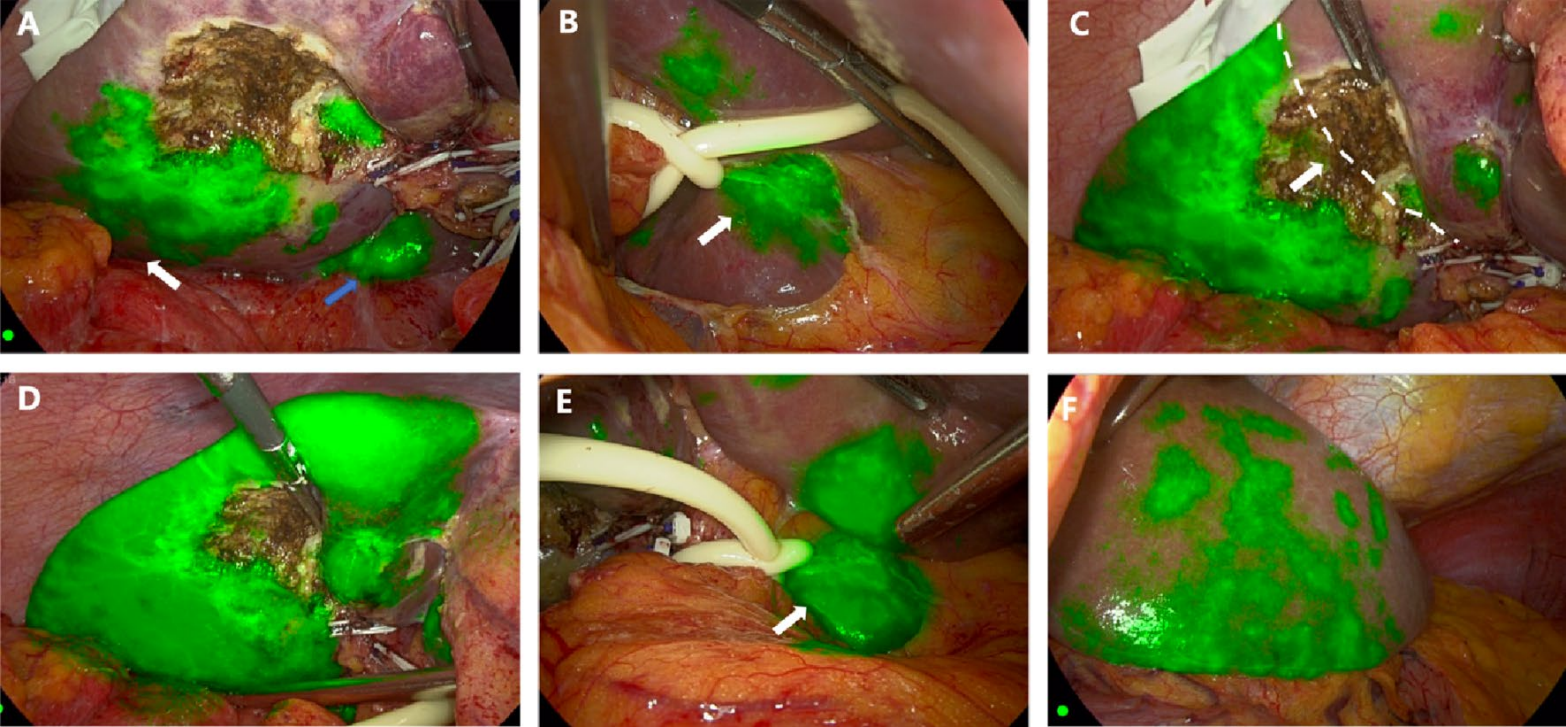
This is an intraoperative video capture of enrolled case 36 (video data in the Video S1 supplement). (**A**) Hepatic fluorescence imaging first appeared in the right posterior lobe (white arrow), caudate process (blue arrow), and left caudate lobe in (**B**) (white arrow); (**C**) As the amount of ICG accumulated in the liver increases, the entire right half of the liver gradually begins fluorescence imaging and demarcates on both sides of the midline of the gallbladder bed, in line with Rex-Cantlie's line11 (white arrow with dotted line). (**D**)–(**F**) Gradually, the left inner lobe left caudal lobe (white joint), and left outer lobe also gradually complete fluorescence development.

**Figure 6:**
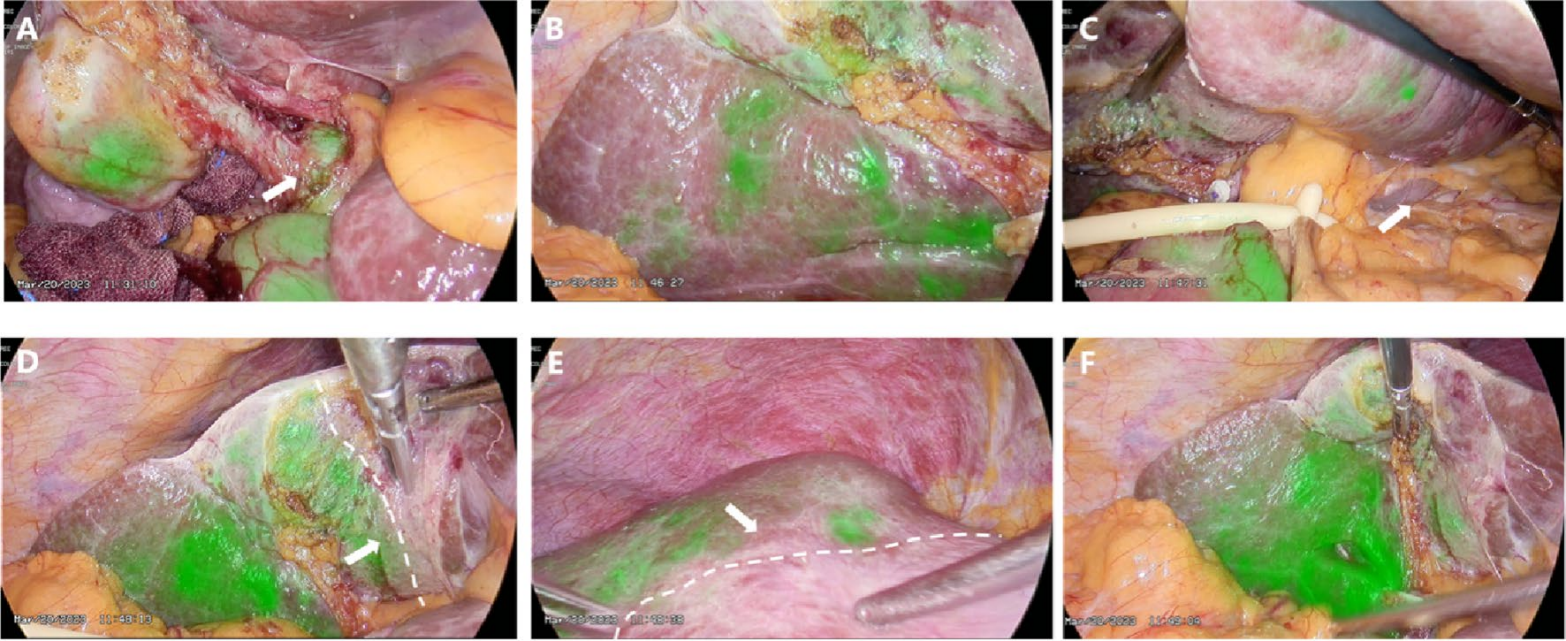
This is an intraoperative video capture of enrolled case No. 38 (video data in supplementary Video S2). (**A**) in the absence of hindrance, bile duct be developed after injection of ICG fluorescence imaging (white arrow) auxiliary safety line LC; (**B**) Progressive fluorescence imaging of the right half liver began; (**C**) The patient had atrophy in the left caudate lobe, and no intraoperative fluorescence imaging was performed. (**D**), (**E**) The fluorescent boundary between the left and right hemispheres of the liver appears (white arrow and dotted line); (**F**) With the increase of ICG over time, fluorescence imaging of the whole liver parenchyma began.

Potential rule of fluorescence development: As shown in Fig. 5, Video S1, and Fig. 6, Video S2, in our study, we not only found that FIT-RB could be used for fluorescence imaging of liver parenchyma but also that this technique of transbiliary tract fluorescence imaging also showed certain rules. We found that the sequence of liver development with this technique was from the “right posterior liver + caudate lobe” to the right anterior lobe to the left liver to gradually complete the fluorescence imaging of the whole liver parenchyma.

**Figure 7:**
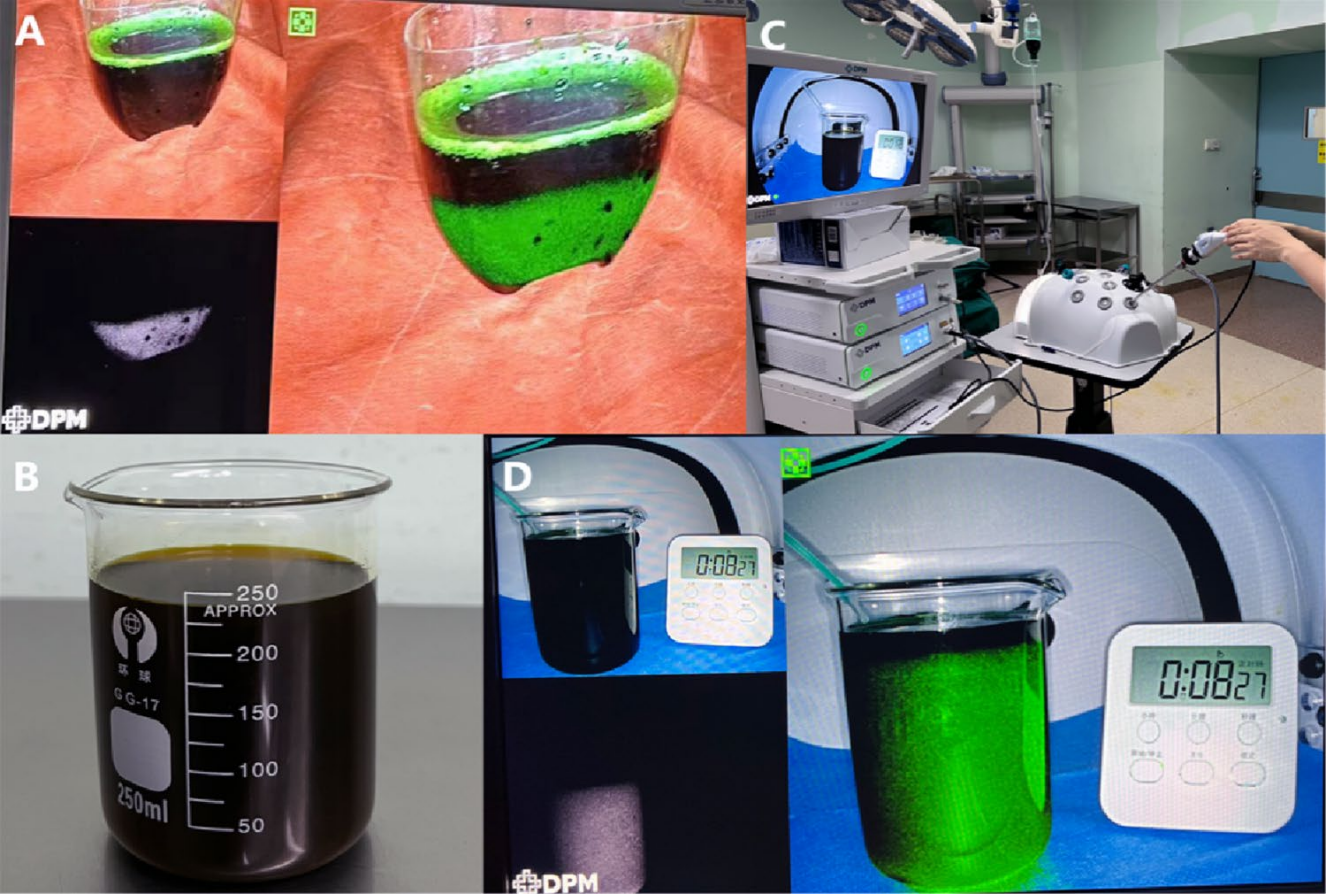
(**A**) 0.5% ICG solution in bile precipitates to the bottom; (**B**) Bile was extracted from ENBD tubes of patients with normal bilirubin. (**C**), (**D**) 0.125% concentration of ICG solution uniformly distributed in bile.

To explore the principle of this development rule, we explore the deposition of different concentrations in normal bile. As shown in the figure below, a 0.5% solution of ICG will completely settle at the bottom with time, proving that it is denser than bile. The 0.125% ICG solution was evenly distributed in the bile. Therefore, we hypothesized that a higher concentration of ICG solution after injection into the biliary tract via ENBD would first enter the relatively lower part of the intrahepatic biliary tract due to gravity (Fig. 7, Video S3) (right posterior − caudate − right anterior + left medial − left external lobe).

## Discussion

Bile duct injury (BDI) is a highly concerning complication of LC, occurring in 0.3–0.7% of cases and resulting in significant perioperative morbidity and mortality, as well as impaired quality of life. Additionally, BDI is associated with high rates of subsequent medico-legal litigation^16^. The principal cause of BDI is typically the misinterpretation of biliary anatomy, which leads to unexpected biliary lesions in 71–97% of cases^17^. To mitigate iatrogenic biliary tract lesions, various methods have been proposed and utilized. One such method that has gained popularity in both elective and emergency settings is near-infrared fluorescent cholangiography (NIRF-C) which effectively highlights biliary anatomy during LC. In 2020, a study by Pesce et al.^18^ found that the technique showed an overall diagnostic accuracy of 86.9% for the gallbladder duct, with no biliary tract injury in the cases studied. Current NIRF-C can help surgeons identify extrahepatic biliary tract anatomy and can reveal abnormal anatomy and accessory biliary duct for safe dissection during laparoscopic cholecystectomy^19,20^.

All 53 patients enrolled in this study underwent LC after ICG injection into ENBD tube and assisted biliary tract imaging, and were discharged successfully without postoperative complications and recovery. However, this technique can only be performed in certain patients with common choledocholithiasis who have undergone ERCP + ENBD. Does not replace the current mainstream ICG fluorescence cholangiography techniques in the management of biliary stones.

To treat patients with limited intrahepatic biliary calculi and choledocholithiasis at stage III of the left liver, our center initially performed choledocholithiasis removal with ERCP + EPBD + ENBD, followed by a second stage of fluorescence laparoscopic and anatomical hepatectomy. “Portal vein-negative staining” was achieved by injecting ICG through the ENBD tube after the intraoperative extrinsic dissection of the segment III liver pedicle for intraoperative fluorescence navigation. Furthermore, we observed that the fluorescence image interfaced with our portal injection ICG fluorescence development exhibits a more durable interface guidance effect without fluorescence drift (Video S4). Building on the success of this trial, we designed a prospective clinical study and achieved a higher success rate of good imaging in patients with low preoperative bilirubin levels. Postoperative LC levels of total bilirubin (TBIL) were inversely correlated with the success rate of imaging, with a 9% decrease in success rate for each unit increase in TBIL (OR: 0.91, 95% CI 0.85–0.98; *P* = 0.0082). Additionally, the levels of ENBD-TBIL (OR: 0.97, 95% CI 0.94–0.99; *P* = 0.0173), ENBD-DBIL (OR: 0.96, 95% CI 0.92–0.99; *P* = 0.0176), and LC-DBIL (OR: 0.89, 95% CI 0.80–0.98; *P* = 0.0156) were also negatively correlated with the success rate of imaging. These findings indicate that lower levels of bilirubin are associated with a higher success rate of imaging, with all *P* values being statistically significant.

The ICG retention test (ICG R15) utilizing the ICG biliary excretion principle is an important indicator of liver reserve function and a significant predictor of postoperative liver failure and mortality^21,22^. An increase in plasma bilirubin levels leads to an increase in ICG R15 due to the accumulation of conjugated bile acids in the bloodstream during cholestasis, which may inhibit the NTCP-mediated uptake of indocyanine green (ICG)^11,12^. Therefore, we hypothesize that NTCP may also play a role in transporting ICG molecules from bile to hepatenchymal cells, and may be influenced by plasma bilirubin, thereby affecting the success rate of good liver imaging in our study.

ICG is a carbocyanine dye with a molecular weight of 775, consisting of two lipophilic polycyclic parts connected by a carbon chain and associated with a sulfate group, imparting some degree of water solubility to the molecule^23,24^. The concentration and nature of the solvent influence the tendency of ICG to form aggregates. In our study, the phenomenon observed may indicate that the properties of dissolution and aggregation of ICG at different concentrations in aqueous solution also exist in bile^25^. Additionally, higher concentrations of ICG (0.5 mg/ml) were found to be more successful than lower concentrations of ICG (0.25 mg/ml, 0.125 mg/ml) in this study (*P* = 0.014), possibly due to the deposition of ICG into the peripheral capillary bile duct at a lower level of the right liver under the influence of gravity, thus achieving intrahepatic transport.

ICG is a water-soluble complex that exhibits strong binding affinity to plasma proteins upon injection into human tissues, and following circulation, it is selectively taken up by hepatocytes through organic anion-transporting polypeptide 1B3 (OATP1B3) and Na+-taurocholate co-transporting polypeptide (NTCP). In normal liver tissues, ICG is excreted via the biliary system, facilitated by multidrug resistance-associated protein 2 (MRP2) expressed on the capillary bile duct^26,27^. Our study achieved hepatic parenchymal fluorescence imaging by “retrograde” injection of ICG into the biliary tract, suggesting a bidirectional ICG transport mechanism in the intrahepatic bile duct epithelial cells^28–31^. However, more research is needed to confirm this hypothesis^28–31^.

### Limitation

The concept of fluorescence imaging technique is indeed a fascinating area of study with promising applications in various fields such as medical diagnosis, drug development, and environmental monitoring. However, it is important to acknowledge that there are several limitations that need to be addressed in order to fully demonstrate the usefulness and scientific evidence of this technique. One of the main limitations of the study is the insufficient data. The sample size of the study may have been too small to draw definitive conclusions about the effectiveness of the fluorescence imaging technique. In addition, the study may have lacked diversity in the samples, which could limit the generalizability of the findings. Further, the data collection process and analysis methods may not have been rigorous enough to provide robust evidence for the efficacy of the technique. Another limitation is the lack of comparison with other imaging techniques. It is important to compare the fluorescence imaging technique with existing methods to evaluate its advantages and limitations. Without such comparisons, it is difficult to determine the unique contributions of the fluorescence imaging technique and its potential impact on the field. Furthermore, the study may have also overlooked other important factors that could influence the effectiveness of the technique, such as the specific characteristics of the imaging equipment, the expertise of the operators, and the environmental conditions. These factors could potentially introduce bias into the results and need to be carefully controlled for in future studies. In light of these limitations, it is clear that further research is needed to address these issues and fully establish the usefulness and scientific evidence of the fluorescence imaging technique. Future studies should strive to increase the sample size and diversity of participants, improve the data collection and analysis methods, and compare the technique with other imaging modalities. Additionally, research efforts should be directed towards investigating the potential impact of other relevant factors on the effectiveness of the technique. Overall, while the concept of fluorescence imaging technique holds great promise, it is essential to recognize the limitations of the existing research and take steps to address these limitations in future studies. By doing so, we can ensure that the technique is rigorously evaluated and its potential impact in various fields is fully realized.

## Conclusions

Hepatic parenchymal fluorescent imaging via retrograde biliary approach is a safe and feasible technique and may have imaging rules in accordance with liver segmentation. A 0.5% concentration of ICG is the appropriate choice for transbiliary liver infection. However, the effect of hepatic parenchyma fluorescence imaging is significantly negatively affected by hepatic cholestasis, i.e. preoperative bilirubin.

### Supplementary Information

Supplementary Video 1.

Supplementary Video 2.

Supplementary Video 3.

Supplementary Video 4.

## Data Availability

The raw data supporting the conclusions of this manuscript will be made available by the corresponding author without undue reservation to any qualified researcher.

## Abbreviations

FIT-RB: Fluorescent imaging technique via retrograde biliary approach
ERCP: Endoscopic retrograde cholangio-pancreatography
EPBD: Endoscopic papillary balloon dilatation
ENBD: Endoscopic nasobiliary drainage
ICG: Indocyanine green
LC: Laparoscopic cholecystectomy
TBIL: Total bilirubin level
DBIL: Direct bilirubin level
BMI: Body mass index
DBD: Diameter of the bile duct
